# F-Deepwalk: A Community Detection Model for Transport Networks

**DOI:** 10.3390/e26080715

**Published:** 2024-08-22

**Authors:** Jiaao Guo, Qinghuai Liang, Jiaqi Zhao

**Affiliations:** School of Civil Engineering, Beijing Jiaotong University, Beijing 100044, China; 23111358@bjtu.edu.cn (J.G.); 22110373@bjtu.edu.cn (J.Z.)

**Keywords:** transportation, urban community detection, deepwalk, travel cost, complex networks

## Abstract

The design of transportation networks is generally performed on the basis of the division of a metropolitan region into communities. With the combination of the scale, population density, and travel characteristics of each community, the transportation routes and stations can be more precisely determined to meet the travel demand of residents within each of the communities as well as the transportation links among communities. To accurately divide urban communities, the original word vector sampling method is improved on the classic Deepwalk model, proposing a Random Walk (RW) algorithm in which the sampling is modified with the generalized travel cost and improved logit model. Urban spatial community detection is realized with the K-means algorithm, building the F-Deepwalk model. Using the basic road network as an example, the experimental results show that the Deepwalk model, which considers the generalized travel cost of residents, has a higher profile coefficient, and the performance of the model improves with the reduction of random walk length. At the same time, taking the Shijiazhuang urban rail transit network as an example, the accuracy of the model is further verified.

## 1. Introduction

In order to achieve a specific level of accessibility and coverage, the urban transportation network planning method typically starts with the central area of the city, chooses the transportation hub as the passenger flow distribution point, and connects the chosen passenger flow distribution point with various levels of lines. However, it is challenging for the transportation network created by the city as a whole to meet the travel demand of inhabitants in different parts of the city at the same time due to the disparities in the scale, population density, and travel characteristics of distinct urban communities. In the event that an urban community is divided, the planning of the transportation network within the community comes first, followed by the consideration of the network connections between the various areas. In addition to fulfilling inhabitants’ travel demands inside various communities, the created network can further enhance the transportation links between towns.

We concentrate on urban spatial community detection techniques in this setting. In the realm of complex networks, community detection algorithms were used in earlier studies on the subjective and qualitative spatial community detection of cities [1,2], including not only the traditional urban community detection of land classification [3] but also traditional networks, virtual space transaction networks such as the Bitcoin network [4], and social networks [5]. At present, novel community detection technologies, such as network refining [6], outlier detection [7], and comparative learning [8], may be applied to urban spatial division in the future, and are worthy of further exploration by scholars.

Overall, there are four primary types of community detection algorithms for complex networks: (1) labeled community detection models that combine nodes and edges; (2) edge-based community detection models; (3) overlapping community detection algorithms; and (4) node-based community detection models. Unfortunately, without taking into account the real circumstances of the city’s population distribution, traffic diversion, etc., these complex network-based community detection algorithms merely abstract the urban space as a complex network, either focusing on the weight of a single node in urban space [9,10] and taking its membership degree as the primary consideration to carry out multi-layer community detection [11,12], or considering the spatial structure of nodes [13,14] in community division, ignoring the travel characteristics of urban residents themselves.

The use of the Word2Vec model by Yao et al. [15] and others to explore the spatial relationship of points of interest (POI) in land use classification is growing as a result of the emergence of geographic data and social big data. Zhang et al. [16] combine high-resolution remote sensing image data and POI data to propose a new urban land use model based on the mutual correlation function (CC-FLU), which solves the potential shortcomings of models using single-source data and fusion methods. Li [17] et al. determined Zhengzhou’s functional zoning by screening the microblog check-in data and processing it using a multi-density spatial clustering technique. In response to arguments made by Niu [18] et al. that the Word2Vec model was unable to adequately capture the spatial heterogeneity among urban communities, the Word2Vec model and the Doc2Vec model were fused; Node2Vec [19] optimized the sequence extraction strategy for the random walking on Deepwalk architecture and various parameter selections are applicable to various types of network data. Yan [20] et al. contend that the urban spatial structure is too complex to be solely represented by natural language sequences. Consequently, they incorporate the spatial distance between points of interest (POIs) into the Word2Vec model and present the Place2Vec model. Other researchers, relying on deep learning algorithms [21,22,23], extract word vector features from various spatial points within the city to achieve urban spatial community detection.

In order to make the community detection model more consistent with the urban spatial structure, some scholars began to study multi-layer transportation networks. A multi-layer traffic network structure can capture the complex interaction between different traffic modes. In the public transportation system, there are a variety of transportation modes (such as buses, subways, taxis, shared bicycles, etc.), each of which has its own unique operating characteristics and coverage. [24,25,26] The multi-layer network model can treat these different modes of transportation as different network layers, so as to more fully reflect the complexity and diversity of the entire transportation system [27,28]. Luo [29] et al. capture latent knowledge from both network topological structure and node attributes through a two-layer representation method. A weighted co-association matrix-based fusion algorithm (WCMFA) is proposed to detect the inherent community structure in attributed networks using multi-layer fusion strategies. Yildirimoglu [30] et al. introduce a novel method to identify active areas or community structures in city networks using multi-layer graphs. Their method processes trip data through Voronoi segmentation, applies community detection per layer, and tests an algorithm to reveal a unified multi-layer community structure. However, in community detection, there are few applications of multi-layer traffic networks, mostly social networks and synthetic networks [31,32,33], which may represent a new research field in the future.

However, these studies have three major shortcomings: (1) The travel regularity and number of residents are not fully integrated into the community detection model. (2) The spatial structure characteristics of urban traffic networks are not fully integrated into the community detection model. (3) In the Deepwalk study, because the RW algorithm is biased towards large-degree nodes in sampling, the samples will not accurately reflect the network topology information, and the random walking sequences improved by Node2Vec do not represent the actual travel routes of residents.

A Deepwalk model is suggested based on the generalized trip costs of the residents in order to close the research gap. Our research is divided into three parts:

(1) The generalized travel cost of residents is incorporated into the RW model.
(2) The improved logit model is used to distribute passenger flow, and the word vector sequence is obtained.
(3) The final sequence of word vectors is input into the Word2Vec model to learn the node embedding, which realizes the community detection of urban space.

The rest of the paper is organized as follows: Section 2 develops a Deepwalk model based on residents’ generalized travel costs; Section 3 contains the model verification; Section 4 presents a case study; and Section 5 presents the conclusions, as well as some suggestions for future research directions.

**Figure 1:**
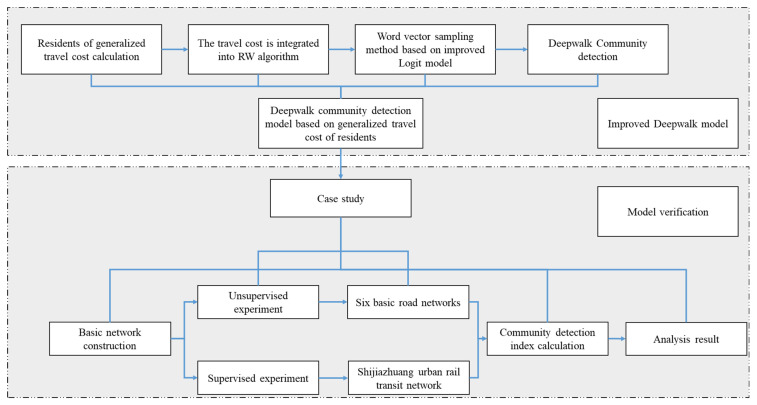
Flowchart of the paper.

Figure 1 describes the logical framework of the research method in this paper in detail, so as to facilitate readers to gradually understand how our research progresses from theoretical conception to practical verification.

## 2. Methods

We are dedicated to building a complete model for community detection in urban space. The Word2Vec model is trained on the incoming sequences by the original Deepwalk model, which uses the RW algorithm to pass the sequence generated by random walking of each node in the network as a natural language string. The Word2Vec model then returns a low-dimensional parameter vector for representation. Consequently, in order for Deepwalk to yield a useful result, the random walk’s length, the sequence it generates, and the number of random walks are all essential.

To this end, we propose a Deepwalk model based on residents’ generalized travel costs, which is divided into three parts: (1) constructing the topology of the urban spatial road network; (2) based on residents’ generalized travel costs, amending the RW algorithm and assigning the passenger flow according to the improved logit model, determining the final sequence of word vectors; and (3) importing the sequence of word vectors into the Word2Vec model to learn node embeddings, use PCA and t-SNE algorithms for dimensionality reduction, and finally divide the community structure of the network by clustering with K-means algorithm.

### 2.1. Urban Transportation Network

Let $G=\left(V,E\right)$ be an urban transportation network, where $V=\left\{v_{1},v_{2},\cdots ,v_{n}\right\}$ denotes the set of large passenger distribution points in *G*, and $E=\left\{\left.\left(v_{i},v_{j}\right)\right|v_{i},v_{j}\in V\right\}$ denotes the set of roads in the network, $\left|E\right|=m$, noting that $A_{n\times n}={\left(a_{ij}\right)}_{n\times n}$ is the corresponding adjacency matrix of *G*. If there exists a road that can go straight through between the passenger distribution points $v_{i}$ and $v_{j}$, then $a_{ij}=1$, otherwise $a_{ij}=0$.

### 2.2. Broad Travel Cost for Residents

When choosing urban public transportation travel, the number of transfers and the degree of resident congestion are key factors affecting residents’ willingness to travel. The more transfers required, the longer the travel time and the more congested the flow of residents, resulting in a lower likelihood of residents choosing that travel path [34]. The time spent by residents on public transportation is divided into in-vehicle travel time and out-of-vehicle waiting time:

(1) $$T_{1}^{r}=\sum_{i=1}^{N^{r}-M^{r}}t_{mi}^{r}+\sum_{i=1}^{N^{r}-1}t_{\left(i,i+1\right)}\;\forall r\in R.$$

where $T_{1}^{r}$ is the in-vehicle time; $t_{mi}^{r}$ is the stopping time at the intermediate station of the *i*th non-interchange station in the path; $N^{r}$ is the total number of stations in the path; $M^{r}$ is the total number of interchanges in the path; and $t\left(i,i+1\right)$ is the travel time in the interval.

The transfer time (waiting time outside the car) for residents is as follows:

(2) $$T_{2}^{r}=\sum_{k=1}^{M^{r}}\left(t_{zk}^{r}+0.5t_{sk}^{r}\right){\left(1+\alpha k\right)}^{\beta }\;\forall r\in R.$$

where $T_{2}^{r}$ is the transfer time, $t_{zk}^{r}$ is the travel time of the *k*th transfer station in the path; $t_{sk}^{r}$ is the departure interval. α and β are the passenger’s transfer sensitivity and correction coefficient of the number of interchanges. The values of parameters are explained in detail in a previous study [34]. Dr. Chai conducted relevant simulation experiments and reached relevant conclusions: a path with more transfer times tends to have more average travel time, and the greater the transfer sensitivity, the more obvious the phenomenon. When α=1, β=2, the convenience and directness of passenger travel are relatively more reasonable

The fare calculation method of urban rail transit is divided into two cases, sectional fare and one-ticket-two-system, and the specific fare is denoted as $f_{sub}$ without considering the special card and special charge cases. In order to unify the fare into the time cost $t_{time}$, a time-cost conversion factor is introduced χ.

(3) $$t_{time}=f_{sub}*\chi .$$

The expression for the time-cost conversion factor A is:

(4) $$\chi =\frac{P_{work}*T_{work}}{GDP}.$$

where $P_{work}$ is the number of people employed in the case city; $T_{work}$ is the number of hours worked in a year; and *GDP* is the annual national *GDP* of the case city.

Excessive passenger flow and interval fullness at a station will affect residents’ travel and lead to an increase in travel costs. We mimic the *BPR* [35] function to define the flow delay coefficient(FDC) φ of station *i* based on the intensity of passenger flow at the next stop of residents, which reflects the impact of passenger congestion on residents’ entry and transfer.

(5) $$BPR:\;t_{i}=t_{0}\cdot \left(1+\gamma_{1}{\left(\frac{Q}{C}\right)}^{\lambda_{1}}\right)\;,\;FDC:\;\varphi =1+\gamma {\left(\frac{v_{i}^{r}}{v_{i-1}^{r}}\right)}^{\lambda }\;.$$

where in *BPR*, $t_{i}$ is the time it takes to actually cross the section, $t_{0}$ is the road-free travel time, *Q* is the volume of traffic passing through the section at that time, *C* is the actual traffic capacity of the road section, $\gamma_{1}$ and $\lambda_{1}$ are undetermined parameters of the model, and the recommended values are 0.15 and 4. In FDC, $v_{i}^{r}$ is the intensity of passenger flow; r and λ are the tuning coefficients; in summary, the generalized travel cost of residents is:

(6) $$C_{se}^{r}=0.5S^{r}\cdot \varphi \;+\;T_{2}^{r}\cdot \varphi \;+\;T_{1}^{r}\;+t_{time}\;=0.5S^{r}\left[1+\gamma {\left(\frac{v_{i}^{r}}{v_{i-1}^{r}}\right)}^{\lambda }\right]+\sum_{k=1}^{M^{r}}\left(t_{zk}^{r}+0.5t_{sk}^{r}\right){\left(1+\alpha M_{k}^{r}\right)}^{\beta }\left[1+\gamma {\left(\frac{v_{k}^{r}}{v_{k-1}^{r}}\right)}^{\lambda }\right]\;+\sum_{i=1}^{N^{r}-M^{r}}t_{mi}^{r}+\sum_{i=1}^{N^{r}-1}t_{\left(i,i+1\right)}+t_{time}.$$

where $C_{se}^{r}$ is the resident’s generalized travel cost, $S^{r}$ is the departure interval of the train when the resident is at the starting point, and its value is equal to $t_{sk}^{r}$ by default, and the resident’s generalized travel cost is an important reference for the subsequent determination of the stochastic wandering path.

### 2.3. Random Walk Path Assignment Based on Residents’ Travel Costs

**Figure 2:**
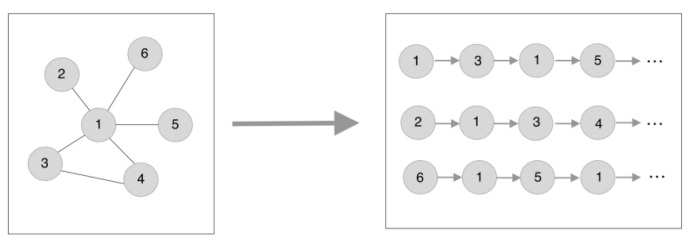
Random walk schematic.

The original random walk algorithm picks network nodes by continuously walking around the network topology with the connecting edges of the nodes [36,37]. Its inputs and outputs are shown in Figure 2.

The number of residents at the node is the number of random walks in the sequence based on the generalized travel costs. Using the improved logit model for passenger flow allocation, the residents will be divided into different paths in accordance with different probabilities. The logit method commonly uses impedance as a constant multi-path passenger flow allocation method, the premise of which is the assumption that each resident is self-perceived to determine the impedance of the road section, and the perceptual impedance is expressed as follows:

(7) $$T_{r}^{ij}=t_{r}^{ij}+\varepsilon_{r}^{ij}.$$

where $T_{r}^{ij}$ is the perceived impedance of path *r* between nodes *i*, *j* of the road network, $t_{ij}^{r}$ is the actual traffic impedance, and $\varepsilon_{r}^{ij}$ is the random error term. The actual traffic impedance can be expressed by the generalized travel cost of residents:

(8) $$t_{r}^{ij}=\theta C_{se}^{r}.$$

According to the definition of utility, the greater the utility, the greater the probability that the resident will choose that path:

(9) $$U_{r}=-T_{r}^{ij}=-\left(t_{r}^{ij}+\varepsilon_{r}^{ij}\right)=-\left(\theta C_{se}^{r}+\varepsilon_{r}^{ij}\right).$$

Assuming that the random error terms $\varepsilon_{se}^{r}$ are independent of each other and follow a Gumbel distribution [38,39], the probability that a resident chooses path *r* is:

(10) $$p_{se}^{r}=\frac{\exp\left(-\theta C_{se}^{r}\right)}{\sum_{q\in R}\exp\left(-\theta C_{se}^{q}\right)\;.}$$

According to the “multiplication” invariance of the logit model, the probability $r_{i}$ that a resident chooses the path $p_{se}^{r}$ can be expressed as follows:

(11) $$P_{se}^{r_{i}}=\frac{\exp\left(-\theta C_{se}^{r_{i}}/\bar{C_{se}}\right)}{\sum_{i=1}^{m}\exp\left(-\theta C_{se}^{r_{i}}/\bar{C_{se}}\right)\;\;.}$$

where $\bar{C_{se}}$ is the average of the generalized travel costs of all random wandering generated sequences between nodes *s* and *e*; *m* is the number of different random wandering sequences generated at node *j*.

Taking node *j* as an example, the specific steps of the algorithm are as follows:

Step 1: Starting from node *j*, generate a randomized wandering sequence of length walk_length and number_walks, which will be denoted as $R=\left(r_{1},r_{2},r_{3},\cdots ,r_{m}\right)$.

Step 2: Calculate the generalized travel cost for each path, denoted as $C_{se}=\left(C_{se}^{1},C_{se}^{2},C_{se}^{3},\cdots ,C_{se}^{m}\right)$, according to Equation (6).

Step 3: Based on the improved logit model, Equation (11) is used to determine the probability of residents choosing each path, denoted as $P_{se}=\left(P_{se}^{1},P_{se}^{2},P_{se}^{3},\cdots ,P_{se}^{m}\right)$.

Step 4: If the number of residents at node *j* is n, then the number of paths A is:

(12) $$Path_{i}=n\cdot P_{se}^{i}\cdot r_{i}.$$

Then the final sequence generated at node *j* is $Finalpath_{j}=\left(Path_{1},Path_{2},Path_{3},\cdots ,Path_{m}\right)$. *l* is the number of road network nodes, and the randomized wandering sequence of the road network *G* is $PathG\;=\;\left(Finalpath_{1},Finalpath_{2},Finalpath_{3},\cdots ,Finalpath_{l}\right)$.

### 2.4. Urban Community Detection Model Based on Improved RW

We use random wandering sequences based on residents’ generalized travel costs to represent the word vectors at the nodes, incorporate the Word2Vec model in the field of NLP for learning, sample the data, use PCA and t-SNE algorithms to reduce the dimensionality, and ultimately divide the community structure of the network by clustering with the K-means algorithm.

#### 2.4.1. Word2Vec Model

There are two main Word2Vec models: the CBOW model and the Skip-Gram model. The Skip-Gram model is utilized to process the samples and update the feature vectors of the nodes to predict their context [40,41].

Each word w in the Skip-Gram model is associated with two learnable *D*-dimensional vectors (i.e., word embeddings), i.e., denoted $u_{w}$ when it is the center word and $v_{w}$ when it is a context word. Given the center word $w_{c}$, the probability of correctly predicting the context type $w_{t}$ is computed by the *Softmax* function:

(13) $$p\left(\left.w_{t}\right|w_{c}\right)=\frac{\exp\left(u_{w_{t}}^{T}v_{w_{c}}\right)}{\sum_{l=1}^{V}\exp\left(u_{l}^{T}v_{w_{c}}\right)\;.}$$

where V represents the total number of words in the predicted library, $u_{w_{t}}$ and $u_{l}$ represent the word vectors for the input and output of the word w. The goal of the Skip-Gram model is to minimize the cross-loss entropy between the predicted probability and the true probability:

(14) $$Minimize\;Z\;=-\frac{1}{T}\sum_{t=1}^{T}\sum_{c=1}^{K}\log p\left(\left.w_{t}\right|w_{c}\right).$$

where K denotes the number of context words corresponding to the size of the set sliding window, and T denotes the number of words in the training set. In order to speed up the training time, we adopt the negative sampling method [42], and the corresponding objective function is as follows:

(15) $$Minimize\;Z\;=-\log\sigma \left(v_{w_{t}}^{T}v_{w_{c}}\right)-\sum_{i=1}^{k}E_{w_{i}\;P_{n}\left(w\right)}\left[\log\sigma \left(-v_{wi}^{T}v_{w_{c}}\right)\right].$$

where K denotes the number of negative examples and $v_{wi}$ denotes the negative example word. Its corresponding probability formula is:

(16) $$P\left(w_{i}\right)=\frac{f{\left(w_{i}\right)}^{3/4}}{\sum_{j=0}^{n}\left(f{\left(w_{j}\right)}^{3/4}\right)\;.}$$

where $P\left(w_{i}\right)$ denotes the probability that word $w_{i}$ is selected as a negative example word, $f\left(w_{i}\right)$ represents the number of occurrences of word $w_{i}$ in the corpus, and n denotes the total number of words in the corpus.

#### 2.4.2. Community Detection Model

This model takes the word vectors processed by the Word2Vec model and reduces the high dimensional data to low dimensions by the PCA algorithm, and then reduces the target data to two dimensions by using the t-SNE algorithm [43,44]. The t-SNE algorithm can map each data point into two-dimensional space, which performs worse than the PCA algorithm when in high dimensions but outperforms the PCA algorithm for low dimensional space, such as three dimensions and two dimensions. The similarity of two original data points in high dimensional space is defined as follows:

(17) $$p_{\left.j\right|i}=\frac{\exp\left(-{\left|x_{i}-x_{j}\right|}^{2}/2\sigma_{i}^{2}\right)}{\sum_{k\neq i}\exp\left(-{\left|x_{i}-x_{k}\right|}^{2}/2\sigma_{i}^{2}\right)\;.}$$

(18) $$p_{\left.j\right|i}=\frac{p_{\left.j\right|i}+p_{\left.i\right|j}}{2N}.$$

where $P\left.j\right|i$ is used to measure the degree of proximity of $x_{i}$ and $x_{j}$, can be regarded as a Gaussian distribution with variance $x_{i}$ around $\sigma_{i}^{2}$. *P*, *j* is the symmetric similarity of the two data points, and the similarity of the mapped points in the 3D space is defined as follows:

(19) $$q_{i,j}=\frac{f\left(\left|x_{i}-x_{j}\right|\right)}{\sum_{k\neq i}f\left(\left|x_{i}-x_{k}\right|\right)}\;with\;f\left(z\right)=\frac{1}{1+z^{2}}.$$

The purpose of the t-SNE algorithm is to minimize the Kullback–Leibler scatter between two similarity matrices computed in different spaces:

(20) $$KL\left(\left.P\right|Q\right)=\sum_{i,j}P_{i,j}\log\frac{p_{i,j}}{q_{i,j}}.$$

After dimensionality reduction using the t-SNE algorithm, clustering is performed using the K-Means algorithm [45], which aims to minimize the squared error *E*:

(21) $$E=\sum_{i=1}^{k}\sum_{x\in C_{i}}{\left|\left|x-\mu_{i}\right|\right|}_{2}^{2}.$$

where $\mu_{i}$ is the mean vector of cluster $C_{i}$, also known as the center of mass, defined as:

(22) $$\mu_{i}=\frac{1}{\left|C_{i}\right|}\sum_{x\in C_{i}}x.$$

#### 2.4.3. Indicators for Model Evaluation

After outputting the clustering results, different metrics are used for judging. For unsupervised experiments, silhouette coefficients are used to verify the accuracy; for supervised experiments, module degree values, silhouette coefficients, Davies–Bouldin coefficients, ARI, AMI, and Harmonic mean values are used to verify the accuracy.

## 3. Model Verification

The default parameters of the original Deepwalk model are used for node embedding of the road network dataset [20], i.e., number_walks = 20, vector_size = 256, walk_length = 8, alpha = 0.03, min_alpha = 0.0007, window_size = 4. For the improved Deepwalk model, number_walks is the number of residents at the node, and α=1, β=2, λ=4,  γ=0.15, θ=1.866 in the stochastic wandering sequence based on the generalized travel cost. Since the initialized cluster centers of the K-means algorithm will have a certain impact on the results, in the experiments, we call the K-means algorithm in ‘sklearn’ and perform the K-means algorithm 50 times for the dataset of each network K-means algorithm and then take the maximum value of the 50 measurements, and the maximum value is taken as the simulation result.

**Figure 3:**
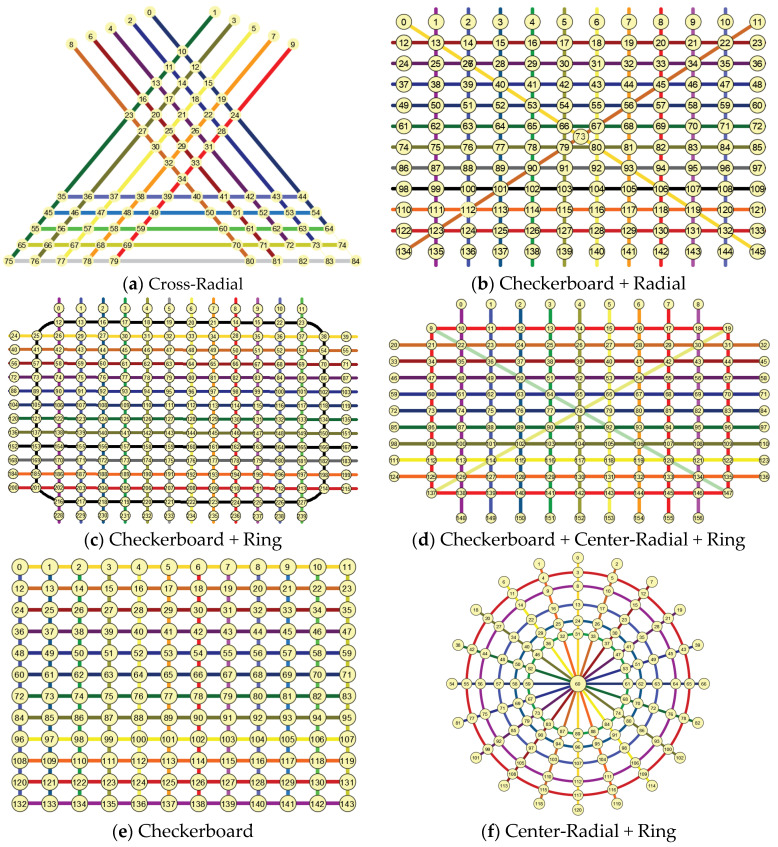
Basic road network pattern.

Although the urban road network structure is inextricably linked to the structural characteristics of the city itself, its form generally consists of one or more combinations of basic road network forms. Therefore, in order to take into account a variety of road network forms, the basic road network forms calculated in this section include Cross-Radial, Checkerboard + Radial, Checkerboard + Ring, Checkerboard + Center-Radial + Ring, Checkerboard and Center-Radial + Ring, as shown in Figure 3.

**Figure 4:**
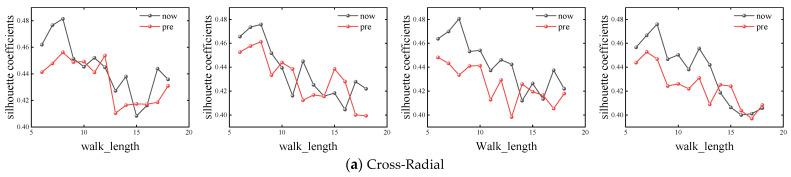

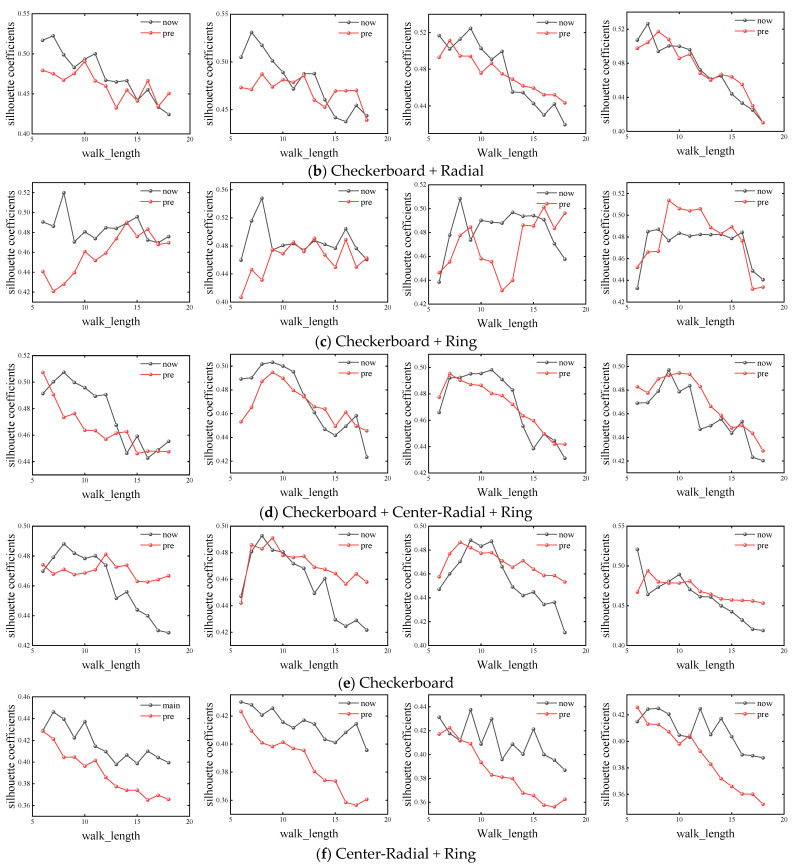
Calculation results of base network silhouette coefficients with different parameters.

In order to ensure the accuracy of the experiment, this paper fixed the number of random walks as number_walks = 10, 15, 20 and 25, and calculated the simulation results of different basic road networks with walk_length from 6 to 18, respectively. The corresponding silhouette coefficients of different road network shapes under different parameters are shown in Figure 4.

It can be seen that when number_walks is smaller than 20, the performance of the F-Deepwalk model is superior to that of the Deepwalk model when walk_length is smaller, except for the checkerboard road network. When walk_length ranges from 8 to 12, the F-Deepwalk model performs better, and its optimal value generally appears when walk_length ranges from 8 to 9. With the continuous increase of walk_length, the contour coefficients of the Checkerboard + Radiate network and Checkerboard network under the Deepwalk model will be reversed, but the maximum value still appears in the F-Deepwalk model. When number_walks is equal to 25, the contour coefficients of the Chessboard + Ring network and Chessboard network reach the maximum value in the Deepwalk model, and the overall performance is better than that of the F-Deepwalk model, but the overall optimal value is still smaller than that of F-Deepwalk model when number_walks is less than 20. Taking the Chessboard + Ring network as an example, the F-Deepwalk model obtains the maximum value when number_walks = 15, walk_length = 8, and its value is 0.54795. The Deepwalk model obtains the maximum value when number_walks = 25, walk_length = 9. Its value is 0.51377, an increase of 6.65%.

**Figure 5:**
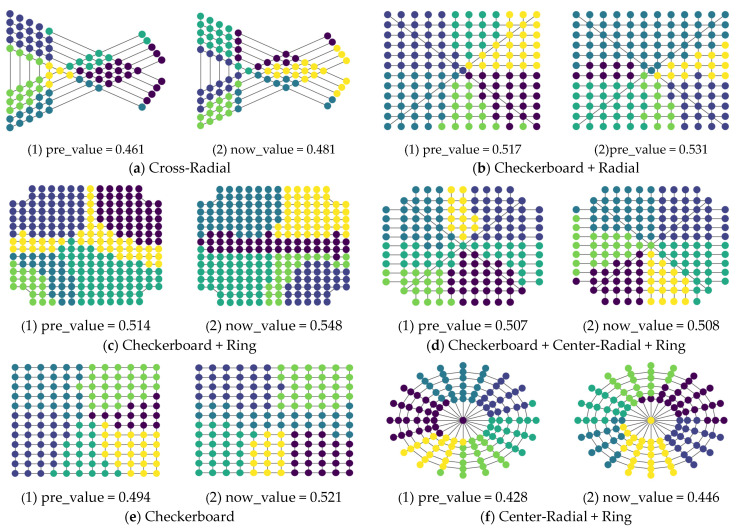
Optimal result division diagram.

In order to more concretely determine the advantages of the F-Deepwalk model, we present the optimal community division results of different models, as shown in Figure 5.

It can be seen that the F-Deepwalk model has no obvious enhancement effect in Checkerboard + Center-Radial + Ring, but has obvious enhancements in other wire network types.

## 4. Case Study

In order to further illustrate the accuracy of the model, we take the Shijiazhuang urban rail transit network as an example, conduct simulation experiments on it, and compare it with the actual urban spatial situation. With the acceleration of urbanization in recent years, Shijiazhuang, the capital city of Hebei Province, has exhibited growing traffic demand. In order to relieve urban traffic pressure and improve citizens’ travel efficiency, Shijiazhuang actively builds and constantly improves its rail transit network. Shijiazhuang rail transit network, with its efficient, convenient, and environmental protection characteristics, has become an important link to connect the city’s various regions. Through scientific and reasonable route planning, the network can effectively cover the main functional areas, commercial areas, residential areas, and transportation hubs of the city, and ensure the on-time rate and operation efficiency of the trains. Passengers can rely on accurate train schedules to arrange travel, reduce waiting time, and greatly facilitate the daily travel of citizens.

**Figure 6:**
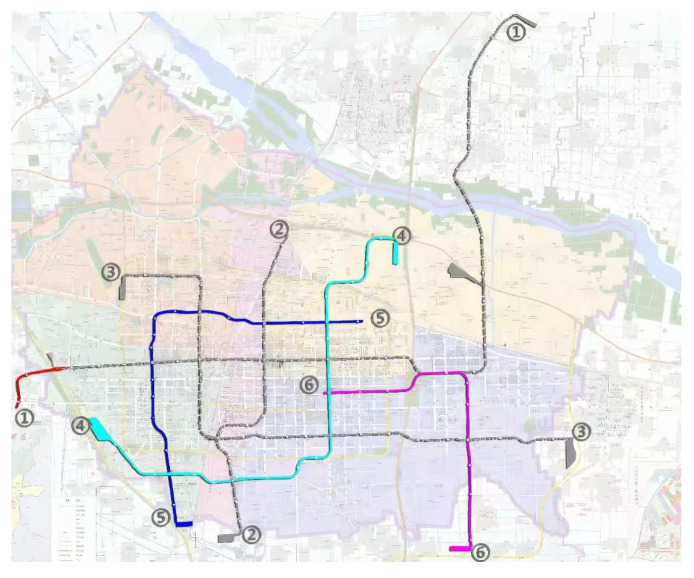
Shijiazhuang urban space community (data source: https://sjz.bendibao.com/traffic/2019218/48216.shtm (accessed on Day 14 Month 7 Year 2024)).

Figure 6 shows the urban space community detection situation and rail transit network topology of Shijiazhuang city, respectively, with different colors representing the different subdivisions in which the stations are located.

**Figure 7:**
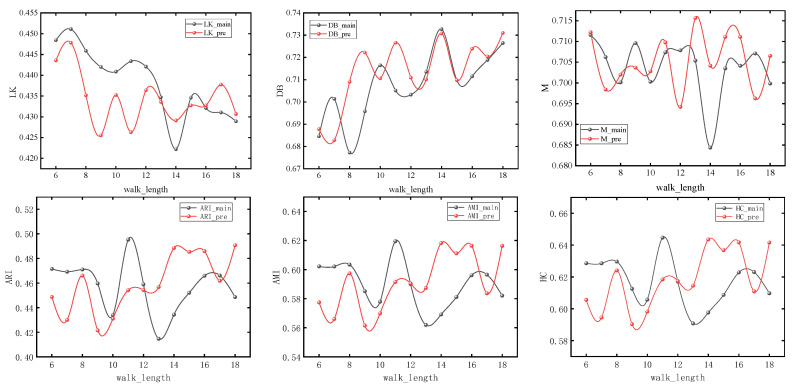
Experimental results under different lengths of random walks.

**Figure 8:**
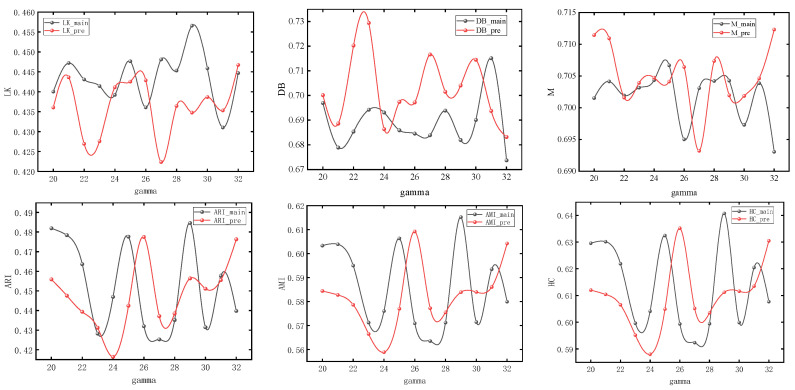
Experimental results under different numbers of random walks.

**Figure 9:**
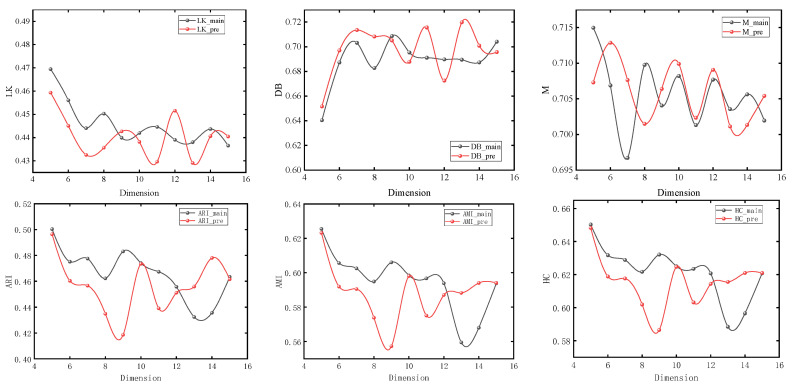
Experimental results under different dimensionality reduction levels.

The nodes with different colors in the figure correspond to the urban spatial communities where the nodes are located in the network. It can be seen from Section 3 that the model usually obtains the maximum value when number_walks = 20. In order to simplify the experiment, we first set number_walks = 20, then adjust walk_length, then fix walk_length and readjust number_walks. In order to ensure the accuracy of the experiment, we also adjusted the dimensionality of PCA reduction. The modularity values (M), silhouette coefficients (LK), Davies–Bouldin coefficients (DB), ARI, AMI, and Harmonic mean values for different parameters are shown in Figure 7, Figure 8 and Figure 9.

It can be seen that with the change of parameters, the overall trend of silhouette coefficients and Davies–Bouldin coefficients of the improved Deepwalk model are better than the original model, and the modularity values are not much different between the two. In addition, the overall trend of the improved model is better than the original model when walk_length, gamma and dimensionality are small, but the performance of the original model is gradually reversed as the parameter values continue to increase. However, the maximum values of ARI, AMI, and HC all appear in the improved model, with maximum values of 0.51283, 0.63679, and 0.66077, which are improved by 4.1%, 3.4%, and 2.1%, respectively, compared with the original model.

## 5. Conclusions and Future Work

### 5.1. Conclusions

We have developed a Deepwalk model based on residents’ generalized travel costs to ensure that the number of random walks and the length of random walks at each node are realistic, and the contributions of this model are as follows:

(1) Based on the original random walk algorithm, the generalized travel costs of residents and the improved logit model are integrated to determine the random walk path that is more in line with the actual demand. At the same time, the PCA and t-SNE algorithms can not only process high-dimensional data efficiently but also find the cluster structure and similarity in the data.

(2) The model is applied to simulate the basic road network configuration. The results show that the F-Deepwalk model is more accurate when walk_length is low, and the results are better than the Deepwalk model in any state.

(3) In order to further verify the accuracy of the model, the Shijiazhuang urban rail transit network is used for experiments. The results show that the model has high accuracy and is helpful in improving the accuracy of urban community division. This paper provides a reference for subsequent network planning.

### 5.2. Policy Suggestion

This model provides an innovative and refined new perspective for urban planning. By using this model to accurately divide urban communities, we can understand the characteristics and needs of different communities more scientifically, so as to guide relevant departments to develop more practical management measures and service programs.

For the transportation authority, after clarifying the boundaries and characteristics of each community, the transportation authority can formulate differentiated traffic flow restrictions according to the specific conditions of different communities. For example, stricter parking management and traffic flow diversion measures should be implemented around communities with dense residents and heavy traffic pressure. In communities with frequent commercial activities and large passenger flow, it may be necessary to optimize road layouts and increase public transportation facilities to alleviate traffic congestion. For the urban planning bureau, based on the results of community division, public resources can be more accurately allocated, such as parks, green spaces, schools, hospitals, etc. By knowing the demographics, age distribution, and income levels of each community, the planning bureau can ensure that the layout of public facilities meets the needs of residents and avoids wasting resources.

### 5.3. Limitations and Future Work

Due to the sampling uncertainty of the random walk algorithm, this paper adopts the method of averaging by multiple simulations to reduce the error, but this still has the problem of unstable experimental results; meanwhile, for cities, it is slightly one-sided to consider only the number of inhabitants, the travel paths of inhabitants and the structure of the urban road network. In the future, on the one hand, we will study a new sampling model to ensure sampling accuracy; on the other hand, we will consider incorporating land value and policy documents of different cities into the sampling sequence to ensure that the results of neighborhood classification are more realistic.

## Data Availability

The raw data supporting the conclusions of this article will be made available by the authors on request.
